# SARS-CoV-2 infection results in a unique lung proteome long after virus resolution in the hamster

**DOI:** 10.1038/s44298-024-00049-x

**Published:** 2024-08-24

**Authors:** Amrit S. Boese, Bryce M. Warner, Peter McQueen, Robert Vendramelli, Nikesh Tailor, Bryan D. Griffin, Mable Chan, Jonathan Audet, Anders Leung, Stuart McCorrister, Chris Grant, Garrett Westmacott, Darwyn Kobasa

**Affiliations:** 1 Special Pathogens Program, National Microbiology Laboratory, Public Health Agency of Canada, Winnipeg, MB Canada; 2 Mass Spectrometry and Proteomics Core, National Microbiology Laboratory, Public Health Agency of Canada, Winnipeg, MB Canada; 3 Department of Medical Microbiology and Infectious Diseases, Max Rady College of Medicine, University of Manitoba, Winnipeg, MB Canada

**Keywords:** SARS-CoV-2, Viral host response, Virus-host interactions

## Abstract

Long COVID or post-acute sequelae of COVID-19 (PASC) remains an ongoing public health issue that causes impairment for those afflicted and diminishes their ability to contribute to society. To address the host response underpinning respiratory PASC, we used the Golden Syrian hamster model infected with ancestral SARS-CoV-2 and examined its lung proteome in a longitudinal experiment. We infected young 6-week old male and female hamsters with 10^5^ TCID_50_ of virus via the intranasal route and sampled the lung at 1, 3, 5, and 31 days post infection (dpi). We compared the infected lung proteome to that of uninfected sex-matched controls. We found almost no differences in protein levels at 1 dpi, with hundreds at 3 dpi, and thousands at 5 dpi. Many overlapping differential protein levels and pathways were seen in both sexes at 3 and 5 dpi including the Coagulation and Complement cascades. Notably, we found differences between the sexes at 31 dpi which included many targets with decreased levels of protein in the males. We also noted an increase in 7 proteins in both sexes at 31 dpi including proteins responsible for airway mucosal layer integrity such as Mucin 5B and Calcium-activated chloride channel regulator 1. Longitudinally, 38 proteins were changed in levels across more than one timepoint in the males but only three proteins were in the females, Secretoglobin family 1 A member 1, Poly [ADP-ribose] polymerase, and Apolipoprotein D. Overall, we show that there are changes to the lung proteome at 31 dpi, a time when no SARS-CoV-2 remains, and that there are sex differences in that proteome after infection with the ancestral strain. We conclude that biological sex should be examined as a variable when testing medical countermeasures for PASC in the Golden Syrian hamster due to host differences between the sexes.

## Introduction

The COVID-19 pandemic has resulted in nearly 7 million deaths globally with at least 750 million surviving infection^1^. Of those that survived, a subset continue to suffer from post-acute sequelae of COVID-19 (PASC)^2^ also known as long COVID, which encompasses a number of neurological and respiratory symptoms^3^. The US CDC estimates that PASC prevalence could be as high as 20% in those aged 18–64 and 25% in 65+^4^, which is a significant sociological and economic burden to society as PASC can prevent people from functioning normally due to an ongoing need for healthcare. Although survival from infection has generally improved from 2021 onwards due to the availability and efficacy of COVID-19 vaccines and therapeutics as well as natural immunity, further insight into the host response after naïve infection is a continued need as there are those that continue to suffer from PASC resulting from the ancestral strain when most of the population was immunologically naïve against SARS-CoV-2. This insight into how host organs are affected past infection resolution is needed to establish potential host-mediated drivers of downstream sequelae, with the hope that they can be targeted for therapeutic intervention. Understanding the host response at a later timeframe is especially important for the ancestral strain of SARS-CoV-2, the dominant strain that infected the population before vaccines or therapeutics were available and may disproportionately result in PASC. Others have specifically shown olfactory abnormalities^5^ and behavioral abnormalities in Golden Syrian hamsters^6^ a month after initial infection with the ancestral strain. Examining the host response in the lungs of the Golden Syrian hamster model, long after the virus has cleared, could show whether there are persisting changes affecting lung homeostasis. For our study, we sampled up to 31 days post-infection (dpi) to determine whether there were host changes at that timepoint that may impact lung homeostasis.

In addition, epidemiological studies demonstrate that females suffer from PASC at a higher rate than males^7–10^, highlighting the need to investigate biological sex as a variable for host response to SARS-CoV-2 infection. A longitudinal analysis of host response stratified by biological sex may shed light on PASC presentation and this information could guide sex-specific therapies for PASC. There is growing evidence that sex biases viral infection outcomes including disease severity^11^ and host immune response, which can be virus-specific [reviewed in^12^]. As an example, although influenza virus and coronavirus both target the respiratory system, influenza results in higher disease severity in females^13^ while MERS-CoV^14^, SARS-CoV^15^ and SARS-CoV-2^16^ results in higher disease severity in males. Preclinical models recapitulate these features for SARS-CoV-2, as we and others have demonstrated sex-related differences in disease severity and underlying host response in the Golden Syrian hamster after SARS-CoV-2 infection, which shows a male sex bias for disease severity^17,18^. The Golden Syrian hamster model exhibits key clinical signs similar to mild to moderate human infection including weight loss, pulmonary viral replication and pathology, plus viral shedding and as it is a non-lethal model, it could serve as a good model for the development of PASC. We previously showed that males did not regain weight as quickly as females, with older males being slower at regaining weight than younger males. Males also shed viral RNA longer than females, with older males shedding for significantly longer than younger males, suggesting impaired infection resolution due to sex and age. Levels of crucial cytokines involved in the immune response in the lungs and blood also had a sex bias with elevated IL-10 and IL-2 mRNA expression detected in females. This sex bias in SARS-CoV-2 infection has also been seen in the ferret model with sex differences in interferon expression and viral burden^19^. We thus stratified biological sex when examining the lung host response in the Golden Syrian hamster model of SARS-CoV-2 infection.

We hypothesized that the lung proteome would have a sex bias with prolonged changes after infection with the ancestral SARS-CoV-2 in Golden Syrian hamsters. As the lungs are the main target of SARS-CoV-2 infection, we examined the proteome of the lung in the SARS-CoV-2 hamster model at 1, 3, 5, and 31 dpi in both sexes using a high volume intranasal inoculation. The proteome was examined using an unbiased, unlabeled mass spectrometry approach. We then compared the proteome from infected animals to sex-matched uninfected controls to determine if there were significant differences in protein levels. We found that the lung proteome was generally similar between infected males and females at earlier timepoints up to 5 dpi, but the proteomes were different at 31 dpi, with many proteins decreased in levels in males. Both sexes had increased levels of proteins that are responsible in maintaining the integrity of the airway mucosal layer at 31 dpi including Mucin 5B and Calcium-activated chloride channel regulator 1 (CLCA-1) indicating a potential ongoing hyperaccumulation or secretion of mucus. Altogether, the evidence indicates that sex should be considered a variable when testing medical countermeasures against PASC.

## Methods

### Animal inoculations and sample collection

All experiments were performed at the National Microbiology Laboratory of the Public Health Agency of Canada under the Animal User Document H-20–006, approved by the animal care committee at the Canadian Science Center for Human and Animal Health according to guidelines set by the Canadian Council on Animal Care. All procedures were performed under inhalation anesthesia using isoflurane. All efforts were made to reduce the number of animals used and to minimize animal suffering. Six-week-old male and female Golden Syrian hamsters were obtained from Charles River Laboratories (Wilmington, DE, USA) and acclimatized for at least one week prior to experiments. Animals were housed in groups of 5 with sexes separated with food and water being provided as desired. Experimenters were not blinded to the experimental groups due to few trained staff for rodent care in high containment. The animals were inoculated through the intranasal route with 100 µL total of ancestral SARS-CoV-2 containing 10^5^ TCID_50_ diluted in DMEM. A group of uninfected males and uninfected females were used for controls. All infectious work was performed in a Biosafety containment level 4 laboratory. The sequence of the viral stock used for experiments is publicly available on GISAID under accession ID EPI_ISL_425177.

Animals were euthanized by first being anaesthetized with inhalation isoflurane followed by cervical dislocation and necropsied on 1, 3, 5, and 31 dpi and the same region of the lung was dissected out at each timepoint to keep the comparison as consistent as possible. The nasal turbinates from 1, 3, and 5 dpi animals were also collected as well as blood for serology from 31 dpi animals. The tissues were flash frozen and kept at –80 °C until further processing for mass spectrometry or TCID_50_ assays.

A second set of archived proximal and distal lung samples from a previous hamster infection were also included in the proteomic analysis. For the archived samples, proximal and distal lung samples were kept frozen at -80°C from 6 week old males and females (*n* = 5). Proximal and distal samples were also taken from two control animals that were inoculated with 100 µL of DMEM only.

### SARS-CoV-2 spike specific enzyme-linked immunosorbent assay (ELISA)

Serum samples were taken from all day 31 post-infection animals and tested for IgG binding in an ELISA described previously^17^ with minor modifications. Briefly, 96-well flat-bottom high-binding microplates were coated with recombinant SARS-CoV-2 spike protein (Acrobiosystems, Newark, DE, USA) diluted in PBS to a concentration of 25 ng per well and incubated overnight at 4°C. The plates were washed four times with PBS + 0.1% Tween 20 and then blocked with 5% skim milk powder in PBS-Tween 20 for 1 hour at 37°C. The plates were then washed again with PBS-Tween 20 before adding PBS diluted serum from 1:200 to 1:6400 in duplicate and plates were incubated overnight at 4°C. After plates were washed with PBS-Tween 20, the secondary antibody AffiniPure Goat Anti-Syrian Hamster IgG (H + L) (Jackson ImmunoResearch, West Grove, PA, USA cat#107–035–142) was added at 1:1000 for one hour at 37°C. After a final PBS-Tween 20 wash, TMB substrate was added and incubated for 15 minutes at room temperature followed by the addition of 1 M Sulfuric acid (H_2_S0_4_) to stop the reaction. The plates were read at OD450nm to obtain measurements.

### TCID_50_ assay for virus quantification

Tissue samples including nasal turbinates and lung samples were kept frozen at -80°C until the TCID50 assay was performed. Ancestral SARS-CoV-2 was tittered using Vero cells that were grown in MEM. Samples were thawed and homogenized in media containing 1% FBS and 1X L-glutamine using a 5 mm stainless steel bead in a Bead Ruptor Elite Tissue Homogenizer (Omni International, Kennesaw, GA, USA). These were then clarified by centrifuging at 1500 *×* *g* for 10 min. Ten-fold serial dilutions were made and in triplicate added to 90–100% confluent cells in a 96 well plate format, which were incubated at 37 °C with 5% CO_2_. The cytopathic effect was measured at 5 dpi and the Reed and Muench method^20^ was used to calculate TCID_50_ value per gram of tissue.

### Preparation of samples for proteomics analysis

One hundred milligrams of lung tissue from each animal was homogenized in 4% SDS containing loading buffer and removed as per biosafety level 4 protocols from containment. The samples were processed for trypsin digestion using the S-Trap mini columns (Protifi, Fairport, NY, USA) as per the manufacturer’s instructions. Briefly, the S-trap was used to remove detergents and other contaminants that interfere with mass spectrometry analysis. Samples were washed and then re-suspended in trypsin (Pierce, 1:10 enzyme:protein) in 50 mM triethylammonium bicarbonate (TEAB, Thermo Scientific) with an overnight on-filter digestion at 37°C. Following trypsin digestion and collection of the peptides, the samples were concentrated to near-dryness under vacuum centrifugation and re-suspended in nano-LC buffer A (2% acetonitrile, 0.1% formic acid).

### LC-MS/MS discovery proteomics data acquisition

Each sample was separately analyzed by liquid chromatography-tandem mass spectrometry (LC-MS/MS), using an Evosep One (Evosep, Denmark) connected in-line to an Orbitrap Exploris 480 mass spectrometer with a nano-electrospray ion source at 2.3 kV and a FAIMS Pro interface with CV at -45V (Thermo Fisher Scientific). The peptide samples were loaded onto Evotips (Evosep) as per manufacturer’s protocol and were loaded and eluted off an AUR3–15075C18 column (IonOpticks, 15 cm × 75 µm, 1.7 µm beads) using Evosep’s 20-samples-per-day whisper method (58-min gradient).

The following settings were used for LC-MS/MS proteomics data acquisition. The precursor scans were acquired in positive mode in the orbitrap with range of 350–1200 m/z at 120,000 resolution. Fragmentation (HCD) scans were done using the targeted MS2 data independent acquisition mode with 45.7-m/z windows spanning the same range as the precursor scan in 19 windows at a resolution set to 30,000.

### Discovery proteomics data analysis and LC-MS/MS targeted data acquisition

Raw mass spectrometry data was analysed using Spectronaut 15 in directDIA mode with default Biognosys Factory settings. Search results were exported from Spectronaut for further analysis using the R programming language. Expression data was median normalized experiment wide using the proDA R package^21^, Principal component analysis (PCA) was performed using the FactoMiner^22^, and factoextra^23^ R packages. For PCA, proteins were filtered to only contain proteins that had no missing values across all samples. Volcano plots were generated using the EnhancedVolcano package^24^. The R package limma was used to find differentially expressed proteins between groups^25^. In short, limma uses a combination of linear models and Bayesian analysis to estimate differential protein expression between groups. For a detailed explanation of the statistical methods used by limma see^26^. The raw data has been deposited to Massive under Dataset MSV000094634 (https://massive.ucsd.edu/ProteoSAFe/static/massive.jsp).

Based on results from the unbiased proteomics analysis, twenty four proteins were selected for further analysis and validation by targeted mass spectrometry analysis. The data from the first Spectronaut analysis was imported into Skyline (https://skyline.ms/project/home/software/Skyline/begin.view) to find a list of potential peptide targets for unscheduled parallel reaction monitoring (PRM) experiments. A final list of 49 peptides belonging to 24 proteins were selected for targeted analysis using PRM mode on the same LC-MS/MS system used for discovery proteomics described above, using the same LC method with the following differences for the acquisition method. The FAIMS source was removed and the ion source was set to 1.9 kV. Precursor scans were acquired in the range of 400–1500 m/z and MS2 scans were acquired at 7500 resolution with isolation window set to 1.6 m/z. The raw PRM data was imported into Skyline for refinement and quantitative analysis. To test for differential protein expression between groups a limma analysis was performed identical to the unbiased analysis.

### Search tool for retrieval of interacting genes/proteins (STRING) analysis for pathways

The differential protein lists of 2-fold change with adjusted p-values of <0.05 were uploaded into the STRING online tool version 11.5 and *Mesocricetus auratus* selected as the host organism^27^. The results for gene ontology (GO) and KEGG pathways were extracted as well as visualizations for the protein interaction networks. Descriptions of the KEGG pathways can be found at https://www.genome.jp/kegg/pathway.html. The upregulated and downregulated protein lists for each infection group were loaded separately to determine up and down regulated pathways. For those lists where no pathways were identified, i.e. the 31 dpi males, the entire list was uploaded to determine whether there were any interacting protein connections.

## Results

### SARS-CoV-2 infection results in a differential proteome at all timepoints tested except 1 dpi and a divergence between males and females at 31 dpi

To examine the host response over time, lung samples were collected from male and female Golden Syrian hamsters at baseline from uninfected animals and at four post-infection sampling timepoints including 1, 3, 5, and 31 dpi from infected animals (*n* = 5). The infected animals were inoculated with the virus through the intranasal route using a total volume of 100 µL, (50 µL each nare), to facilitate upper and lower airway infection. We confirmed infection in the early timepoint animals by detecting high infectious viral titers in the lungs and nasal turbinates of 1, 3, and 5 dpi animals (Supplementary Fig. 1A, B). To establish that the 31 dpi animals were infected, we examined their serum for the presence of IgG against SARS-CoV-2 spike protein by ELISA. All animals displayed seroconversion, with each animal registering high serum titers of ≥6400. The respective lung samples were homogenized in SDS buffer and processed for mass spectrometry using an on-column trypsin digestion procedure.

Overall, 3235 proteins were detected with 100% coverage across all groups. The dataset was examined for the presence of SARS-CoV-2 nucleocapsid, spike, and ORF3 peptides, which were identified as above baseline in 1 dpi and 3 dpi samples but not at 5 dpi and 31 dpi (Supplementary Fig. 1C–E) while membrane protein was detected as above baseline in 1 dpi, 3 dpi, and 5 dpi samples. This was as expected as viral RNA^17^ and titers peak before 5 dpi (Supplementary Fig. 1A, B).

**Fig. 1:**
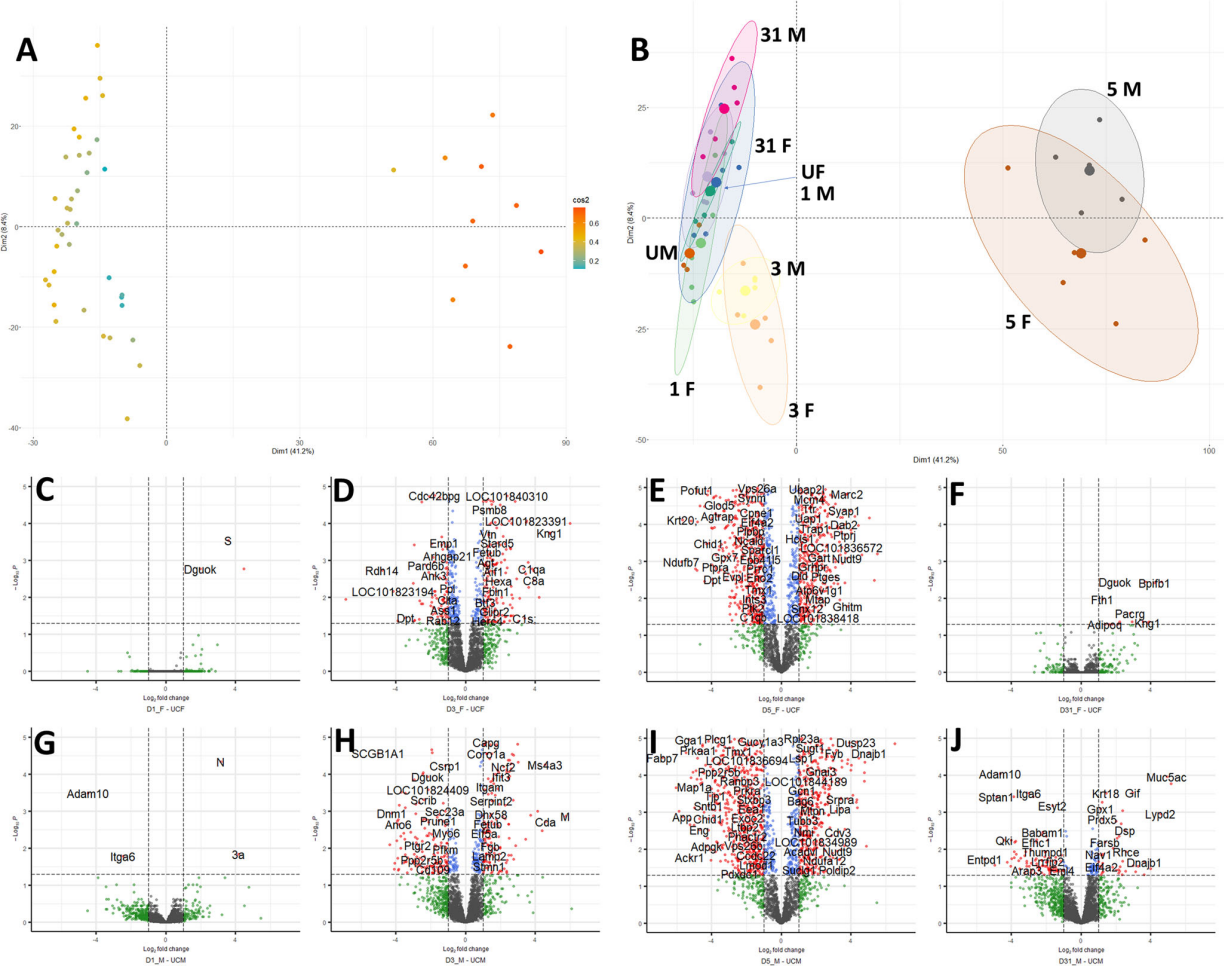
Overview of the proteomic data of the uninfected and infected groups by principal component analysis (PCA) and volcano plots of head-to-head group comparisons for differentially changed proteins. **A** PCA of individual animals (**B**) PCA with groups encircled light green = 1 dpi female (1 F), purple = 1 dpi male (1 M), orange = 3 dpi female (3 F), yellow = 3 dpi male (3 M), brown = 5 dpi female (5 F), black = 5 dpi male (5 M), blue = 31 dpi female (31 F), pink = 31 dpi male (31 M), green = uninfected female (UF), and dark orange = uninfected male (UM); (C)-(J): volcano plots for intergroup comparisons (**C**) 1 dpi female versus control (**D**) 3 dpi female versus control (**E**) 5 dpi female versus control (**F**) 31 dpi female versus control (**G**) 1 dpi male versus control (**H**) 3 dpi male versus control (**I**) 5 dpi male versus control (**J**) 31 dpi male versus control.

We performed a principal component analysis (PCA) on the proteome of all hamsters to determine the relationship of infected to control proteomes (Fig. 1A, B). The uninfected and the 1 dpi proteomes generally clustered together far from the 5 dpi group, notably in the Dim1 axis, and also, to a much lesser extent, apart from the 3 dpi group in the Dim1 and Dim2 axes. The 31 dpi male group had minimal overlap and sat further up the Dim2 axis compared to the other groups. The male and female uninfected groups were relatively similar.

To determine the effects of infection on the lung proteome, we used volcano plots to visualize differences between infected and sex-matched control groups (Fig. 1C–J). At 1 dpi, there was almost no difference between infected and control (Fig. 1C, G). The majority of changes were at 5 dpi (Fig. 1E, I) followed by 3 dpi (Fig. 1D, H), then 31 dpi (Fig. 1F, J). The number of significantly changed proteins was generally similar between male and female at each timepoint with the exception of 31 dpi, where there were more proteins changed in the males, 87, than the females, 15 (Supplementary Table 1).

**Fig. 2:**
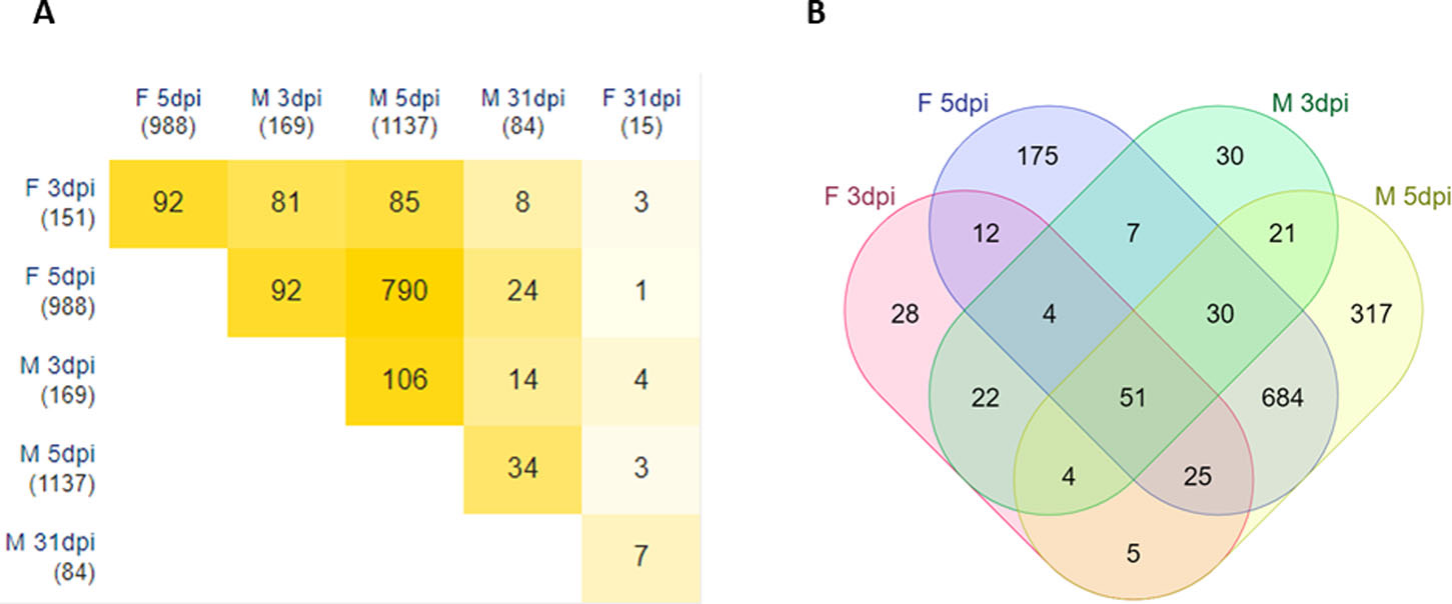
Longitudinal analysis of proteins changed in levels due to infection. **A** All timepoints and groups compared to each other for overlapping proteins; (**B**) Venn diagram of overlapping proteins from 3 and 5 dpi males and females. F = female; M = male

We went on to explore proteins in the infected groups that were ≥ ±2.0-fold with an adjusted *p* value < 0.05 when compared to their respective control (Supplementary Table 1). The 5 dpi groups had the greatest number of protein levels changed compared to uninfected, with 999 in the females and 1148 in the males. The 3 dpi groups had many changes as well but to a much lesser degree than 5 dpi, with 152 in the females and 171 in the males. Perhaps not surprisingly due to temporal proximity, 3 and 5 dpi had many overlapping proteins (Fig. 2A, B) while 31 dpi had a number of unique proteins detected only at that timepoint (Fig. 2A). The 31 dpi groups had 15 changed proteins in females and 84 in males, which was the first timepoint to have a divergence between sexes as females showed a quicker return to baseline.

Longitudinally, the infected males had 9 proteins that were changed at 3, 5, and 31 dpi, where available the known functions of these proteins in the lungs are highlighted (Table 1). A subset of these were significantly changed in females as well and in the same direction of change. In addition to these, there were 25 proteins that were commonly changed between the 5 dpi and 31 dpi sample timepoints and 5 proteins between 3 dpi and 31 dpi (Supplementary Table 2).

**Table 1:** Proteins that were changed in levels at 3 dpi, 5 dpi, and 31 dpi in infected males

	Mean log2 Fold Change			
Protein (Gene)	3 dpi	5 dpi	31 dpi	Potential role in lungs or disease association
Allograft inflammatory factor 1 (Aif1)	2.86 F: 3.01	2.09 F: 2.73	–2.30	Increased in Olfactory bulb of SARS-CoV-2 infected hamsters^45^
Specifically androgen-regulated gene protein isoform X1; X2 (CUNH1orf116)	–3.19	–4.35 F: –2.06	–2.99	None found
Glia maturation factor gamma (Gmfg)	–2.70	6.49 F: 6.36	–2.24	.Mediates neutrophil and T lymphocyte migration via regulation of actin cytoskeletal reorganization^46,47^
LIM and calponin homology domains-containing protein 1 isoform X7; X10; X9; (Limch1)	–2.73 F: -2.5	–3.84 F: –2.6	–3.30	None found
Epidermal growth factor receptor kinase substrate 8-like protein 2 (LOC101830673)	–2.07	–2.50	–2.18	None found
Phosphatase and actin regulator 4 (Phactr4)	–2.95	–3.17	–2.12	In mice, interacts with actin and protein phosphatase 1^48^
Protein scribble homolog (Scrib)	–3.07	–4.23 F: –3.47	–2.06	Lung cell morphogenesis^49^
Delta-sarcoglycan (Sgcd)	–4.52 F: –2.07	–5.95	–3.26	Airway responsiveness^50^
Transmembrane protein 100 (Tmem100)	–3.80	–4.65 F: -3.66	–2.56	Lung endothelial cells with roles in angiogenesis and vascular morphogenesis and integrity^51^

*F* female.

### Network interaction pathways were identified at 3 and 5 dpi with coagulation and complement cascades upregulated at both timepoints

**Fig. 3:**
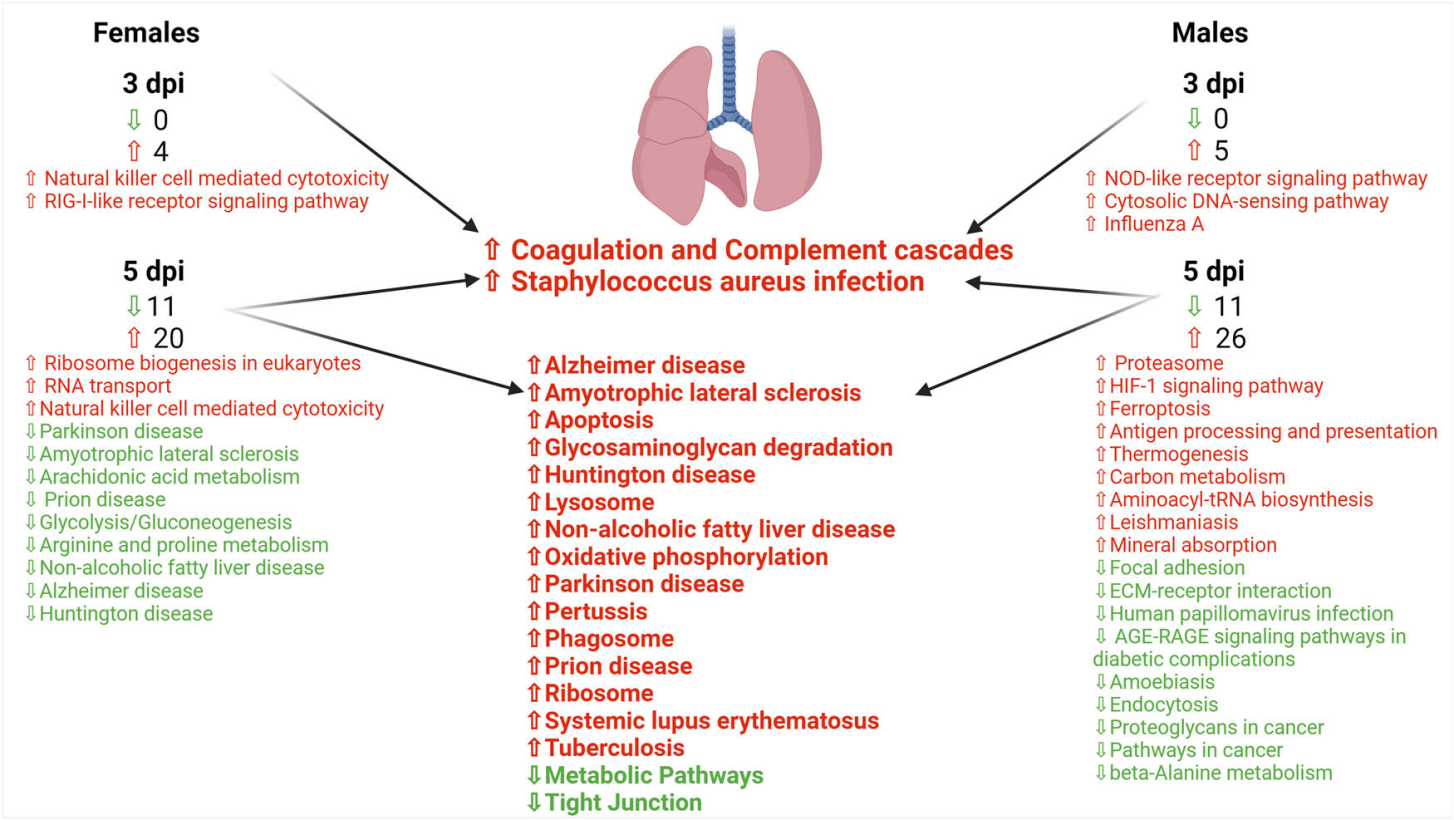
The KEGG pathways identified in the proteome of 3 and 5 dpi males and females and overlap between infected groups (center list) along with those unique to each indicated. The central list closest to the lungs are pathways found in all 3 and 5 dpi males and females while the center lower list are those pathways in common at 5 dpi regardless of sex. Red indicates upregulation of pathway and green indicates downregulation. [Created with BioRender.com].

To establish which representative pathways were associated with the differential proteins within each group, we used STRING analysis, which uses a combination of metrics to establish networks of interacting proteins and identifies classical Kyoto Encyclopedia of Genes and Genomes (KEGG) gene ontology pathways. No pathways were detected in the 1 or 31 dpi groups; however, the 3 and 5 dpi groups had many representative pathways detected (Supplementary Table 3; Fig. 3). The 3 dpi groups had only upregulated pathways with 4 in the females and 5 in the males, while the 5 dpi groups had the most pathways represented with 11 downregulated in each sex, 20 upregulated in females, and 26 in males.

At 5 dpi, there were 17 upregulated pathways and 2 downregulated pathways in common between males and females; however, sex differences were evident at this timepoint as well with unique pathways identified in each. In the males, there were 18 unique pathways with 9 upregulated and 9 downregulated, while the females had 12 unique pathways with 3 upregulated and 9 downregulated (Fig. 3). The common downregulated pathways in both sexes were Metabolic Pathways and Tight Junction. Metabolic pathways had a large number of proteins represented with 74 in the males and 61 in the females, showing that SARS-CoV-2 infection has a considerable effect on metabolism in the lung while fighting infection. Pathways that we found that were also detected in the lung proteome of infected hamsters by others included Ferroptosis, Tight Junction, Mineral Absorption, Beta-Alanine metabolism, Arginine and Proline metabolism, Focal Adhesion, ECM-receptor interaction, and HPV infection^28^. There were quite a number of unique pathways in both sexes at 5 dpi. Some that potentially stand out with relevance to PASC development or conditions associated with them such as diabetes include downregulation of Glycolysis/Gluconeogenesis (map00010) in the females and downregulation of pathways associated with cancer formation in the males (map05200 and map05205). Males at 5 dpi had increases in host responses such as thermogenesis (map04714), carbon metabolism (map01200), and mineral absorption (map04978).

There were 51 proteins in common that were altered in levels at 3 and 5 dpi in both sexes (Fig. 2B), these included Myxovirus resistance protein 1 and 2 (MX1, MX2), 2’-5’ oligoadenylate synthase 1 and 2 (OAS1, OAS2), ubiquitin-like protein ISG15, RNA helicase, Cytochrome P-450, and Granzyme-like proteins. The MX2 protein was increased over 5 fold in the infected groups at both timepoints while both OAS1 and 2’-5’ OAS1A-like were increased by nearly 4 fold, indicating the activation of the innate immune response.

### SARS-CoV-2 results in a unique lung proteome long after infection is resolved

Sampling at 31 dpi revealed a number of changed proteins although a STRING network analysis did not find significant interactions between them. This may indicate that the proteins are independently affecting homeostasis or that their network links in the lung are not yet established in databases used for such analysis. Interestingly, there were 7 upregulated proteins detected in both males and females at the 31 dpi timepoint that were not detected at any other timepoint sampled (Table 2). A number of these proteins have functions in the mucosal layer and the innate immune response, and their increased levels suggest a potential mucin hyperaccumulation or hypersecretion state in both sexes long after viral clearance has been achieved.

**Table 2:** Proteins that were changed in levels at 31 dpi in both sexes

Protein (gene)	Mean log2 Fold Change	Function	Disease association
Mucin 5AC (Muc 5AC) Mucin 5B (Muc 5B)	F – 3.40 M – 5.14 F – 4.81 M – 7.86	Prevent pathogen infection of airway and regulate cellular transcription and signaling^52^	BALF and serum levels are correlated with the development interstitial lung disease^53^ Muc 5AC overexpression protects mice from influenza^54^
Dynein light chain roadblock (Dynlrb2)	F – 3.90 M – 3.54	Forms part of the cytoplasmic dynein complex^55^	Essential for Murine Leukemia Virus trafficking and nuclear entry^56^
Bactericidal/Permeability-increasing-fold-containing family B member 1 (BPIFB1)	F – 3.75 M – 6.55	Innate defense genes^57^ Expressed in respiratory tract of air-breathing vertebrates^58^ Loss diminishes mucociliary clearance in the lungs^59^	Induced in mouse tracheal epithelial cells during Influenza A infection^60^
Calcium-activated chloride channel regulator 1 (Clca1)	F – 3.57 M – 6.72	Controls mucus production in the lungs and regulates innate immune responses^61^	Induced in the lungs by Th2 cytokines^61^
Intelectin-1a-like (LOC101824891 LOC101838992)	F – 2.91 M – 4.37	ITLN1 recognizes β-D-galactofuranose, a galactose present in microorganisms^62^	Expressed in goblet cells in untreated asthma^63^ ITLN-1 is increased in the airways of IL-13-overexpressing mice, component of mucus associated with intense eosinophilic airway inflammation^64^
Parkin coregulated gene protein (Pacrg)	F – 2.72 M – 3.17	NFkB response to TNF^65^ Axoneme assembly^66^	None identified to date

*F* female, *M* male.

The males at 31 dpi had more proteins changed than females and 73.6% of proteins were decreased in levels compared to uninfected controls. A few notable proteins were Galectin-9 (FC –5.04), Coagulation factor III (FC –2.50), Niemann-Pick C1 protein (FC –2.41), and RNA helicase (FC –3.32). Galectin-9 has shown a protective role in humanized ACE2 mice when administered therapeutically after SARS-CoV-2 infection^29^.

**Fig. 4:**
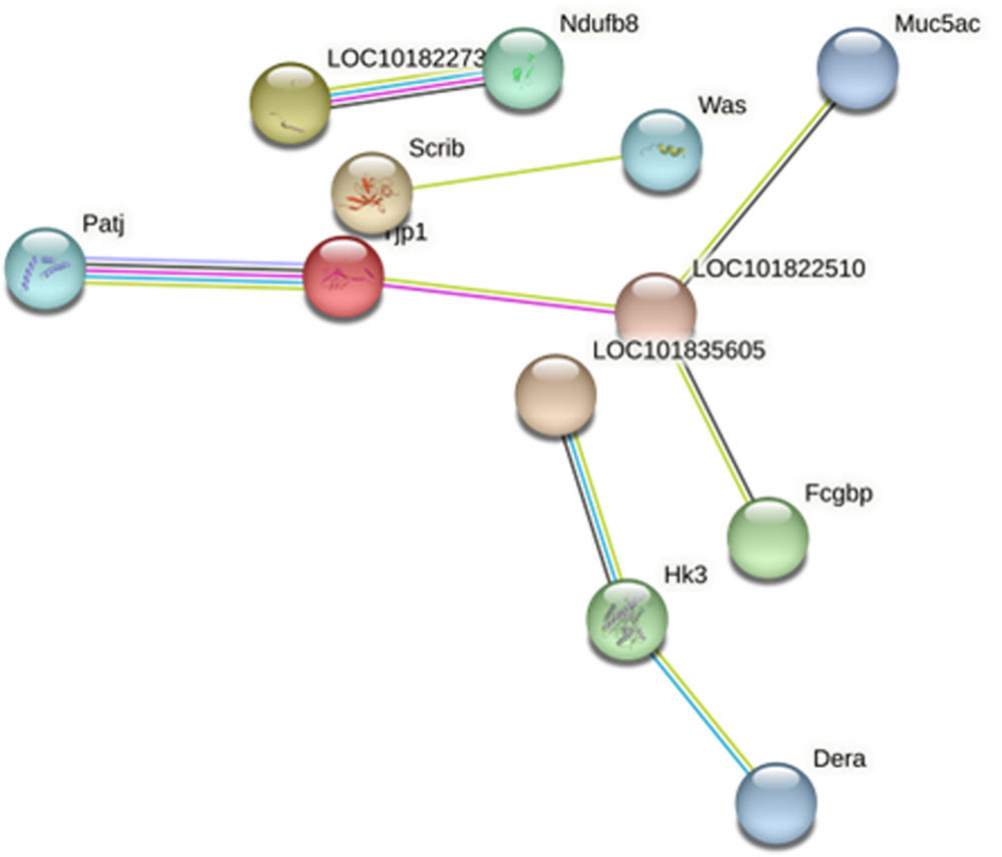
Network analysis of significantly changed proteins in 31 dpi males. STRING network analysis of all 31 dpi changed proteins in the males demonstrates known and predicted interactions present. Circles (nodes) show gene IDs with known interactions represented by dark pink lines (experimentally determined) and light blue lines (curated databases), predicted interactions are indicated by red, black, purple, dark green, dark blue, and light green lines.

Although no pathways were identified at 31 dpi, there were a number of interactions noted between upregulated proteins Mucin 5AC, Calcium activated-chloride channel regulator 1, Fc Gamma Binding Protein (Fcgbp) and downregulated proteins tight junction (tjp) and (Patj) in the males (Fig. 4). In the females, SCGB1A1, also known as Uteroglobin, was increased at 31 dpi (FC 2.40) and 5 dpi (FC 2.46) but was decreased at 3 dpi (FC –2.53). This protein was only detected in the males as decreased at 3 dpi (FC –5.55). The Peptidoglycan-recognition protein (Pglyrp1) was decreased in the females at 31 dpi (FC –2.21) and not changed at any other timepoint.

### Validation using archived samples and targeted mass spectrometry

A second set of archived lung samples collected from mock (DMEM) or infected hamsters at 5 dpi from our previously published study^17^ were prepared and run in an unbiased proteomics experiment to compare to the longitudinal 5 dpi dataset. Although we only detected a total of ~1800 proteins in the archived samples, we did find 36 deregulated proteins in common in the males and 13 in the females between the archived samples and this experiment (Table 3). Between all four datasets, male and female at 5 dpi, there were 2 proteins increased (LOC101841064, Rplp1) and 5 decreased (Cgn, Chchd3, LOC101825465, LOC101836251, Myo6). A complete list of the protein coverage for the datasets can be found in Supplementary Table 4.

**Table 3:** Proteins that were validated using archived samples (5 dpi) or targeted mass spectrometry (3, 31 dpi)

Sex	dpi	Gene ID	Protein	Mean Log2 Fold Change	*p* value
Female	3	Mx2	Myxovirus resistance protein 2	2.51	4.68E–04
	5	Actb	Actin, cytoplasmic 1;Actin, cytoplasmic 1	2.29	2.68E–02
	5	LOC101841064	60 S ribosomal protein L38	2.73	1.35E–02
	5	Chchd3	MICOS complex subunit MIC19 isoform X2;MICOS complex subunit MIC19 isoform X1	–4.80	6.01E–03
	5	Pdha1	Pyruvate dehydrogenase E1 component subunit alpha	–2.64	1.96E–02
	5	Hpx	Hemopexin	2.43	8.44E–03
	5	F11r	Junctional adhesion molecule 1	–2.71	1.66E–02
	5	Cgn	Cingulin	–3.35	1.17E–02
	5	Myo6	Unconventional myosin-6	–2.47	2.97E–02
	5	Fth1	Ferritin	1.87	4.13E–02
	5	LOC101825465	Transmembrane emp24 domain-containing protein 4	–2.43	5.08E–02
	5	LOC101836251	Plectin isoform X19;plectin isoform X13;plectin isoform X11	–1.73	3.83E–02
	5	Myzap	Myocardial zonula adherens protein isoform X1;myocardial zonula adherens protein isoform X2	–1.94	5.08E–02
	5	Rplp1	60 S acidic ribosomal protein P1	1.87	2.68E–02
	31	LOC101822510	Calcium-activated chloride channel regulator 1	5.93	1.91E–03
Male	3	Hpx	Hemopexin	2.71	1.65E–05
	3	Mx2	Myxovirus resistance protein 2	3.17	2.65E–04
	5	Ahsg	Alpha-2-HS-glycoprotein	1.65	7.18E–03
	5	Alcam	Activated leukocyte cell adhesion molecule	–1.80	1.72E–02
	5	Apmap	Adipocyte plasma membrane-associated protein	–3.35	3.70E–03
	5	Cgn	Cingulin	–4.20	2.00E–03
	5	Chchd3	MICOS complex subunit MIC19 isoform X2;MICOS complex subunit MIC19 isoform X1	–5.37	1.94E–03
	5	Col4a1	Collagen alpha-1(IV) chain	–6.35	1.99E–03
	5	Col4a3	Collagen alpha-3(IV) chain	–4.46	1.56E–02
	5	Fth1	Ferritin	2.89	3.25E–03
	5	Hpx	Hemopexin	5.34	3.49E–06
	5	Hrg	Histidine-rich glycoprotein	2.18	1.29E–02
	5	Hspa9	75 kDa glucose-regulated protein	1.52	2.25E–02
	5	Jup	Junction plakoglobin	–1.64	1.16E–02
	5	LOC101825465	transmembrane emp24 domain-containing protein 4	–2.76	3.06E–02
	5	LOC101828182	Serotransferrin	2.04	6.14E–03
	5	LOC101830930	C4a anaphylatoxin	2.39	2.02E–03
	5	LOC101836251	Plectin isoform X19;plectin isoform X13;plectin isoform X11	–2.51	4.48E–03
	5	LOC101838193	interferon-inducible GTPase 1-like	1.86	3.38E–02
	5	LOC101841064	60 S ribosomal protein L38	2.47	1.55E–02
	5	Lama3	Laminin subunit alpha-3	–1.96	1.09E–02
	5	Lamc2	Laminin subunit gamma-2	–2.03	1.73E–02
	5	Lims1	LIM and senescent cell antigen-like-containing domain protein	–1.75	8.45E–03
	5	Lin7c	Protein lin-7 homolog	–4.58	1.73E–02
	5	Lman1	Protein ERGIC-53	–3.41	5.43E–04
	5	Mx2	Myxovirus resistance protein 2	3.77	1.55E–02
	5	Myo6	Unconventional myosin-6	–2.77	1.72E–02
	5	Ndufa9	NADH dehydrogenase [ubiquinone] 1 alpha subcomplex subunit 9, mitochondrial	1.61	4.10E–02
	5	Pafah1b2	Platelet-activating factor acetylhydrolase IB subunit beta	1.56	4.31E–02
	5	Patj	InaD-like protein	–1.74	4.72E–02
	5	Prkca	Protein kinase C	–2.69	1.55E–02
	5	Rplp1	60 S acidic ribosomal protein P1	1.69	3.53E–02
	5	Slc9a3r2	Na(+)/H(+) exchange regulatory cofactor NHE-RF	–2.42	8.05E–03
	5	Tnpo1	Transportin-1	–3.27	4.11E–02
	5	Tns1	Tensin-1 isoform X5;tensin-1 isoform X2	–1.67	3.06E–02
	5	Tpm1	Tropomyosin alpha-1 chain isoform X5	–2.27	1.40E–02
	5	Wars	T1-TrpRS	1.97	1.32E–02
	5	Xdh	Xanthine dehydrogenase	1.92	1.52E–02
	31	Bpifb1	BPI fold-containing family B member 1	5.05	2.99E–05
	31	LOC101838992	Intelectin-1a-like	7.74	2.99E–05
	31	Muc5ac	Mucin-5AC	6.01	1.60E–04
	31	LOC101822510	Calcium-activated chloride channel regulator 1	6.00	9.43E–04

Due to the lack of hamster compatible immunoreagents, especially for many of these relatively exploratory host proteins, we used a targeted mass spec approach to validate a subset of proteins that were deregulated in the 3 and 31 dpi samples. We ran a targeted mass spectrometry for proteins for which peptide sequences were available to determine if we obtained the same results within our samples using this approach. As with our unbiased mass spectrometry experiment, CLCA-1 was significantly increased in both sexes at 31 dpi with 5.93-fold in females (*p* value = 0.0019) and 6.00-fold in males (*p* value = 0.0009). In addition, the 31 dpi males had detectable increases in Mucin 5AC (6.01-fold, *p* value = 0.0002), BPIFB1 (5.06-fold, *p* value = 2.988e–05), and intelectin-1a-like (7.74-fold, *p* value = 2.988e–05). At 3 dpi, both sexes had significant increases in Hemopexin, C3, Serpina1, and MX2 proteins and only the males had increased levels of intelectin-1a-like and scribble (Table 3). The other proteins were not significantly different than controls.

## Discussion

Our study sheds light on respiratory PASC by finding host changes in the lungs at 31 dpi in a younger cohort hamster model that demonstrates mild to moderate COVID-19. In terms of biological sex, we found some overlapping proteins increased in both sexes at 31 dpi; however, differences were also seen including more proteins having decreased expression in the males. In terms of addressing PASC prevalence being higher in human female COVID-19 cases, we did identify disruption in the lung-relevant SCGB1A1 at 31 dpi which was not changed in males. It is also possible that the neurological type of PASC, which we did not measure in our study, is the dominant type present in females. Future work examining potential alterations to lung function will be vital in establishing whether the proteins we detected affect oxygen exchange rates, lung capacity and elasticity, or extracellular remodeling. Our 3 and 5 dpi results add to previous findings that show similar changes at 4 dpi in an aged mixed-sex model^28^. We detected almost no changes to the lung proteome at 1 dpi in either sex.

Our lack of detection of significant changes at 1 dpi is in line with findings that early viral events elude host detection and response including innate immunity due to the lack of recognizable viral motifs and sequestration of viral components during replication. It is possible that one aspect of this delay in detection of the host response is accumulation of cell death caused by the virus which takes some time to occur resulting in a delayed response^30^. This host response is evident at 3 dpi when there were hundreds of proteins significantly changed and thousands by 5 dpi in both sexes. There were 2 upregulated pathways in common in 3 dpi and 5 dpi, Complement and Coagulation cascades (map04610) and *Staphylococcus aureus* infection (map05150). In agreement with our results, both pathways were detected previously in the lung proteome of older mixed sex SARS-CoV-2 infected hamsters despite a different isolate being used^28^. Unique to our females was the upregulated RIG-I-like receptor signaling pathway (map04622) at 3 dpi and Natural killer cell mediated cytotoxicity pathway (map04650) at 3 and 5 dpi. Differences in natural killer cell response between the sexes has also been noted in chronic hepatitis infection^31^ as well as differential expression upon stimulation despite similar total counts^32^, which could in part explain why this pathway was found only in females during SARS-CoV-2 infection. At 3 dpi, the males had unique pathways including NOD-like receptor signaling pathway (map04621), Cytosolic DNA-sensing pathway (map04623), and Influenza A (map05164). At 5 dpi, Focal adhesion (map04510) was uniquely downregulated in the males though it was previously identified overall in the mixed sex study^28^. In our case we used a much younger cohort where it appears to be stratified to the males only. The metabolic pathway also showed up in our 3 and 5 dpi groups and studies on the plasma metabolome in PASC patients shows disruption 2 years after initial infection^33^.

We unexpectedly found 5 upregulated neurodegenerative disease pathways at 5 dpi in both sexes including Amyotrophic lateral sclerosis, Parkinson disease, Huntington disease, Alzheimer’s disease, and prion disease. A protein critical in these pathways, Sod2, was upregulated in both males and females, and this has been detected previously in infected hamster lungs^28^ and the bronchoalveolar lavage fluid (BALF) of patients^34^. Mutations in Sod2 have been associated with lung diseases such as COPD indicating the vital nature of this protein in the lungs^35^. Notably, the females also had downregulation in the neurodegenerative disease pathways while the males did not. This suggests there may be bidirectional effects in the female nervous system in responding to the infection compared to males, which could affect lung homeostasis as well as nervous system signaling to a greater extent in females.

A number of proteins involved in the interferon-stimulated genes pathway were increased in our infected animals including MX2 and OAS1. The induction of these is triggered by the release of type I and III interferons^30^. Studies have shown that MX2 mRNA is elevated in COVID-19 patients, both in nasopharyngeal swabs^36^ and in BALF of mild cases but not severe cases^37^, suggesting that an early induction of MX2 is associated with milder infection. Sencio et al.^38^ found MX2 mRNA elevated in the lungs of infected hamsters at 2, 4, and 7 dpi using a similar inoculum volume as our study and induction was seen in the lungs and BALF of a rhesus macaque model at 3 dpi with a return to baseline by 14–17 dpi^39^. In ferrets, a model that also demonstrates mild SARS-COV-2 infection, MX2 mRNA was elevated in female animals at 2 dpi but returned to baseline by 5 dpi^40^. OAS1 and OAS1A-like proteins which we found to be elevated are in the family of OAS1 innate immune signaling proteins found to decrease COVID-19 disease severity in patients that have a Neanderthal OAS1 isoform^41^. Mutations in OAS1 or OAS2 result in impaired functioning of these proteins, and have been found in a small number of pediatric patients who have progressed to multi-inflammatory syndrome (MIS-C) after initially having COVID-19^42^. Put together, the data suggest that this early OAS and MX2 induction likely helps keep the SARS-CoV-2 infection mild to moderate in the hamster model.

Importantly, we found that the 31 dpi proteome of both males and females was different and that the males had a substantial amount of proteins downregulated. Mucin 5AC protein was increased in the males at 31 dpi, and is known to increase significantly in the lungs of mouse models of asthma when exposed to small particulate matter^43^, suggesting that SARS-CoV-2 infection results in lasting increase in this insult-responsive protein in the lungs.

To validate our data in a meaningful way, we used targeted mass spectrometry for a subset of proteins as immunological reagents were not readily available for our host species to run Western blots. Further refinements could be made to the targeted method including developing selected reaction monitoring assays (SRM) for the target proteins that usually provide quantitative results at an increased sensitivity compared to PRM. Although Western blots tend to be the gold standard for validating mass spectrometry experiments, we found that we could validate substantially more proteins using targeted mass spectrometry and in a quantitative way. There have been recent calls to change from western blotting as a method to validate mass spectrometry data due to the semi-quantitative nature and unreliability of primary antibodies used for detection^44^.

Caution must be undertaken translating our findings from animal to human, especially as experimentally we can use a high titer with direct inoculation. Although the hamster model is a good representation of mild-to-moderate human infection, it needs further validation for human PASC. Nevertheless our findings demonstrate that there are persisting changes in host protein levels in the lungs after SARS-CoV-2 resolution with an ancestral strain in a younger animal cohort. Validating our findings is that the protein expression changes that we detected during acute infection have been observed by other groups^28^. Previous studies looking at a similar timeframe in the hamster model has shown mechanical hypersensitivity in the peripheral nervous system and behavioral changes^5,6^. Overall, as we gain further insights into PASC in humans, refinements in animal models will help lead to a better model. For now, our study adds to the evidence that the Golden Syrian hamster model of disease shows changes in multiple host proteins one month post-infection. Future studies can inform whether these changes are long-lasting and how they may be impacting PASC.

## Data Availability

The proteomics data has been deposited to Massive (https://massive.ucsd.edu/ProteoSAFe/static/massive.jsp) under dataset MSV000094634.

## Supplementary informatiom

Supplementary Table
Supplementary Fig
