# Supplementary material for: SARS-CoV-2 infection results in a unique lung proteome long after virus resolution in the hamster
Source: Npj Viruses. 2024 Aug 24;2:40. doi: 10.1038/s44298-024-00049-x (PMC11721347; doi:10.1038/s44298-024-00049-x)
Supplement: Supplementary file 2 — Supplementary Fig [file 44298_2024_49_MOESM2_ESM.pdf]

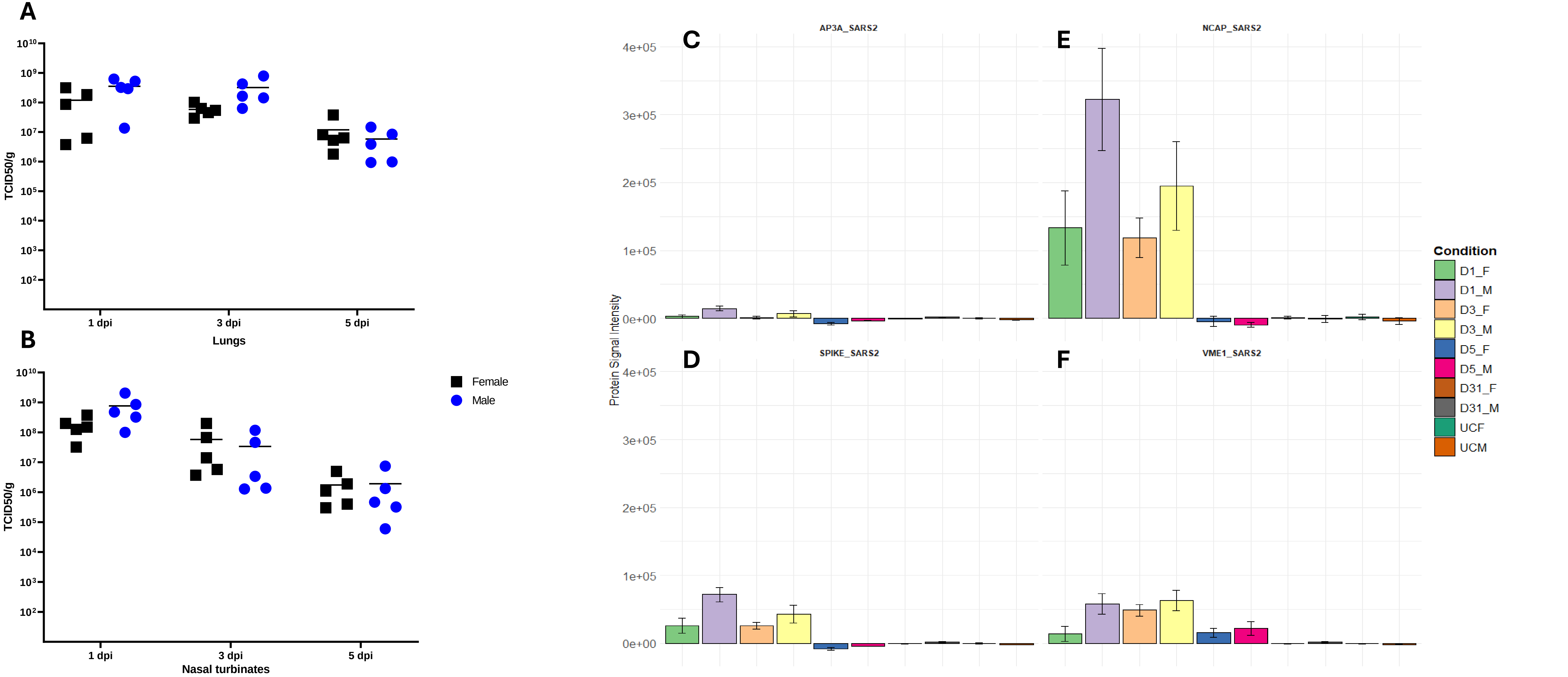

Supplementary Figure 1. Viral titers and peptides in SARS-CoV-2 infected hamsters. Infectious virus present in 1, 3, and 5 dpi females and males in A) lung tissues and B) nasal turbinates; SARS-CoV-2 viral peptides detected by mass spectrometry in the females and males at 1 dpi (D1_F and D1_M), 3 dpi (D3_F and D3_M), 5 dpi (D5_F and D5_M), and 31 dpi (D31_F and D31_M) C) ORF3a protein (AP3A), D) Spike protein, E) Nucleocapsid (NCAP), and F) Membrane protein (VME1). F = female, M = male, UCF = uninfected female, UCM = uninfected male.
